# Prenatal Exposure to Benzophenone-3 Impairs Autophagy, Disrupts RXRs/PPARγ Signaling, and Alters Epigenetic and Post-Translational Statuses in Brain Neurons

**DOI:** 10.1007/s12035-018-1401-5

**Published:** 2018-11-06

**Authors:** Agnieszka Wnuk, Joanna Rzemieniec, Jakub Staroń, Ewa Litwa, Władysław Lasoń, Andrzej Bojarski, Małgorzata Kajta

**Affiliations:** 1 Institute of Pharmacology, Department of Experimental Neuroendocrinology, Laboratory of Molecular Neuroendocrinology, Polish Academy of Sciences, Smetna Street 12, 31-343 Krakow, Poland; 2 Institute of Pharmacology, Department of Experimental Neuroendocrinology, Polish Academy of Sciences, Smetna Street 12, 31-343 Krakow, Poland; 3 Institute of Pharmacology, Department of Medicinal Chemistry, Polish Academy of Sciences, Smetna Street 12, 31-343 Krakow, Poland

**Keywords:** Benzophenone-3, BP-3, Neuronal cell, miRNA, Autophagy, Prenatal exposure

## Abstract

The UV absorber benzophenone-3 (BP-3) is the most extensively used chemical substance in various personal care products. Despite that BP-3 exposure is widespread, knowledge about the impact of BP-3 on the brain development is negligible. The present study aimed to explore the mechanisms of prenatal exposure to BP-3 in neuronal cells, with particular emphasis on autophagy and nuclear receptors signaling as well as the epigenetic and post-translational modifications occurring in response to BP-3. To observe the impact of prenatal exposure to BP-3, we administered BP-3 to pregnant mice, and next, we isolated brain tissue from pretreated embryos for primary cell neocortical culture. Our study revealed that prenatal exposure to BP-3 (used in environmentally relevant doses) impairs autophagy in terms of BECLIN-1, MAP1LC3B, autophagosomes, and autophagy-related factors; disrupts the levels of retinoid X receptors (RXRs) and peroxisome proliferator-activated receptor gamma (PPARγ); alters epigenetic status (i.e., attenuates HDAC and sirtuin activities); inhibits post-translational modifications in terms of global sumoylation; and dysregulates expression of neurogenesis- and neurotransmitter-related genes as well as miRNAs involved in pathologies of the nervous system. Our study also showed that BP-3 has good permeability through the BBB. We strongly suggest that BP-3-evoked effects may substantiate a fetal basis of the adult onset of neurological diseases, particularly schizophrenia and Alzheimer’s disease.

## Introduction

The ultraviolet (UV) absorber benzophenone-3 (2-hydroxy-4-methoxybenzophenone, oxybenzone, 2OH-4 MeO-BP, or BP-3) is the most extensively used chemical substance as a UV filter, especially (but not only) in various personal care products [1]. Additionally, it is a plastic and textile ingredient as well as a component of inks and lacquers mainly used for protection from sun-induced fragility. The production and consumption of BP-3 is enormous; it has been identified that European-only production reaches 100–1000 metric tons per year and shows an upward trend in response to the increasing demand for health protection against skin cancer [2]. In September 2017, according to scientific reports, the European Union Commission has limited the use of BP-3 from 10 to 6% in cosmetic sunscreen products [3]. In 2017, the US Centers for Disease Control and Prevention (CDC) demonstrated that approximately 97% of people are exposed to BP-3 [4].

Prenatal and early postnatal exposures to BP-3 seem to be undeniable. Current data provide evidence that BP-3 easily crosses through the placental barrier since it has been observed in amniotic fluid, placental tissue, cord blood, and fetal blood in research studies with human participants [5–7]. Moreover, the course of pregnancy has been shortened by BP-3, and suboptimal fetal growth has been revealed after maternal exposure to BP-3 [8–10]. Even after birth, newborns and infants are exposed to BP-3 since this chemical has been identified in human breast milk samples from Switzerland and Spain [11, 12]. Furthermore, the blood-brain barrier (BBB) does not seem to be an obstacle for BP-3 either; accumulated BP-3 has been demonstrated in post-mortem adult brains [13]. Additionally, prenatal BP-3 exposure has been linked to improper migration of enteric neural crest cells during embryogenesis, resulting in Hirschsprung’s disease in offspring [14]. Despite the fact that BP-3 exposure is extremely widespread and affects prenatal features, knowledge of the impact of BP-3 on the development of the nervous system is negligible.

The mechanism of neurogenesis (either during neural development or in the adult brain) is complex. It requires homeostasis of different signaling pathways and is tightly controlled by epigenetic and post-translational modifications. The histone deacetylase superfamily is a class of enzymes consisting of histone deacetylases (HDAC) and sirtuins. They are primarily responsible for removing acetyl groups from an amino acid on a histone, thus permitting the transcription of DNA. Moreover, sirtuins are also regulators of metabolic pathways, DNA repair, and the stress response. Small ubiquitin-like modifiers (SUMOs) are proteins that take part in post-translational modifications primarily to provide protein stability. An impairment or disruption of embryonic/postnatal neural development contributes to the majority of neuropsychiatric disorders (e.g., autism, schizophrenia, attention-deficit hyperactivity disorder, depression, and bipolar disorder). Furthermore, recent studies indicated that dysfunction of neonatal neurogenesis is associated with the etiology of neurodegeneration, e.g., Parkinson’s disease [15].

Autophagy is a process of cellular digestion of toxic cytoplasmic material (e.g., misfolded proteins or dysfunctional organelles), and it is a mechanism that provides energy during starvation or cellular stress. Autophagy also plays an essential role in the central nervous system during neurogenesis, and its dysregulation is associated with the etiology of neurodevelopmental and neural degeneration [16]. Autophagy is regulated by a series of autophagy-related genes (*Atg*) and other crucial factors (such as BECLIN-1, MAP1LC3, or AMBRA1). Autophagy is important during the development and maturation of axons, dendrites and synapses. Knockout mice without *Atg5*, *Atg7*, *Beclin-1*, or *Ambra1* exhibit early lethality and neurodegeneration. Mice lacking *Atg7* in the central nervous system (CNS) revealed abnormalities in the cerebral and cerebellar cortices, indicating that autophagy is responsible for the homeostasis of neural cells [17]. Moreover, embryonic disruption of autophagy has an adverse impression on adult neurogenesis throughout the lifespan when adult neurons exhibit dramatic aging [18].

Nuclear receptors participate in a majority of life-dependent processes, including embryogenesis, neural development, and lipid metabolism. For most of the non-steroid nuclear receptors (class II nuclear receptors), the retinoid X receptor (RXR) is an obligatory heterodimerization partner. In mammals, RXRs are encoded by three distinct genes located on different chromosomes—RXRα, RXRβ, and RXRγ. RXRα has been identified primarily as a heterodimerization partner of the majority of nuclear receptors [19]. Studies have shown that retinoid signaling is implicated in the health and disease of the nervous system. Knockout mice with RXRα or RXRβ deficiencies are embryo-lethal, and RXRγ-knockout mice show dysfunction in oligodendrocyte differentiation, spatial learning, and memory function [20, 21]. Moreover, dysfunctional retinoid signaling has been involved in cognitive impairments, schizophrenia, and depression [22–24]. One of the heterodimerization partners of RXR is peroxisome proliferator-activated receptor gamma (PPARγ), which takes part in a wide-range of cellular processes, including lipid and glucose metabolism, apoptosis, and autophagy. PPARγ is mainly expressed in fat tissue and the brain.

The present study aimed to explore the mechanisms of prenatal exposure to BP-3 in neuronal cells, with particular emphasis on autophagy and nuclear receptor signaling as well as the epigenetic and post-translational modifications occurring in response to BP-3. To observe the impact of prenatal exposure to BP-3, we administered BP-3 to pregnant mice, and next, we isolated cells from pretreated embryos for primary neocortical culture. The influence of BP-3 on the autophagic process was assessed by the expression of autophagy-related factors (BECLIN1, ATG7, NUP62, MAP1LC3A, MAP1LC3B), as well as based on the detection of autophagosomes. The impact of BP-3 on RXRs and PPARγ was analyzed via measurement of specific mRNA and protein levels in neocortical cells (by qPCR, ELISA, and western blot). Additionally, to establish the involvement of prenatal BP-3 on microRNA (miRNA) expression, microarray and qPCR analyses were employed. Epigenetic and post-transcriptional modifications were assessed by measuring activity including histone acetyltransferase (HAT), HDAC, sirtuins, and sumoylation. Overall, the effect of prenatal exposure to BP-3 was also evaluated by microarray analysis of the expression profiles of neurogenesis-related genes and neurotransmitter receptors. BBB permeability for BP-3 was assessed by the BBB Kit™ and mass spectrophotometry.

## Methods

### Materials

B27 and Neurobasal media were obtained from Gibco (Grand Island, NY, USA). L-Glutamine, fetal bovine serum (FBS), N-acetyl-Asp-Glu-Val-Asp p-nitro-anilide (Ac-DEVD-pNA), dimethyl sulfoxide (DMSO), HEPES, CHAPS, ammonium persulfate, BP-3, TEMED, TRIZMA base, Tween 20, DL-dithiothreitol, Nonidet NP-40, sodium deoxycholate, protease inhibitor (EDTA-free), bromophenol blue, 2′,7′-dichlorofluorescein diacetate, RIPA buffer, protease inhibitor cocktail for mammalian tissues, Histone Acetyltransferase (HAT) Activity Assay Kit, Histone Deacetylase (HDAC) Assay Kit, Autophagy Assay Kit, and poly-ornithine were obtained from Sigma-Aldrich (St. Louis, MO, USA). Bradford reagent, SDS, 30% acrylamide, 0.5 M Tris-HCl buffer, 1.5 M Tris-HCl gel buffer, Mini-PROTEAN TGX Precast Gels, and Laemmli sample buffer were purchased from Bio-Rad Laboratories (Hercules, CA, USA). 2-Mercaptoethanol was obtained from Carl Roth GmbH + Co. KG, (Karlsruhe, Germany). Immobilon-P membranes were purchased from Millipore (Bedford, MA, USA). The BM chemiluminescence western blotting substrate (POD) was purchased from Roche Diagnostics GmbH (Mannheim, Germany). ELISA kits for BECLIN-1, NUP62, MAP1LC3A, MAP1LC3B, RXRα, RXRβ, RXRγ, and PPARγ were purchased from Shanghai Sunred Biological Technology Co. (Shanghai, China). The culture dishes were obtained from TPP Techno Plastic Products AG (Trasadingen, Switzerland). The anti-BECLIN-1 rabbit polyclonal antibody (sc-11427), anti-NUP62 mouse rabbit polyclonal antibody (sc-48373), anti-MAP1LC3α/β mouse monoclonal antibody (sc-398822), rabbit polyclonal anti-RXRα antibody (sc-774), mouse monoclonal anti-RXRβ antibody (sc-56869), mouse monoclonal anti-RXRγ antibody (sc-514134), anti-PPARγ mouse polyclonal antibody (sc-7273), and anti-β-actin mouse monoclonal antibody (sc-47778) were purchased from Santa Cruz Biotechnology, Inc. (Santa Cruz, CA, USA). Rabbit monoclonal anti-ATG7 antibody (26313) was obtained from Cell Signaling Technology (Danvers, MA, USA). The RNeasy Mini Kit, miRNeasy Mini Kit, miScript II RT Kit, miScript miRNA PCR Arrays, and RT^2^ Profiler PCR Arrays were obtained from Qiagen (Valencia, CA, USA). The High Capacity cDNA-Reverse Transcription Kit, the TaqMan Gene Expression Master Mix, and TaqMan probes for specific genes encoding *Hprt*, *Becn1*, *Nup62*, *Atg7*, *Map1lc3a*, *Map1lc3b*, *Rxrα*, *Rxrβ*, *Rxrγ*, *Pparγ*, *SNORD95*, *miR-19b*, *miR-33*, *miR-489*, and *miR-509* were obtained from Thermo Fisher Scientific (Waltham, MA, USA). The Global Protein Sumoylation Assay Kit was purchased from Abcam (Cambridge, UK), and the Sirtuin Activity Assay Kit (Fluorometric) was from BioVision (Milpitas, CA, USA). The BBB Kit™ was purchased from PharmaCo-Cell Company Ltd. (Nagasaki, Japan).

### Animals and Treatment

In this study, 12 pregnant Albino Swiss mice (Charles River Laboratories, Sulzfeld, Germany) at 7–16 days of gestation were housed individually in a controlled environment (i.e., 21 ± 1 °C, 40–50% humidity, water, and food—ad libitum; natural sequence of day and night—12 h of light and 12 h of darkness with lights on at 7 a.m.) as previously described [25, 26]. Pregnant mice were administered for 10 days, once a day with 50 mg/kg BP-3 at 7–16 days of gestation as subcutaneous injections. The chosen dose was environmentally relevant and did not cause any visible unwanted effects. A single whole human body application of sunscreen cream (2 mg/cm^2^ of cream) provides 40 g for an average body area of 2.0 m^2^ [27]. Until September 2017, the maximum authorized concentration of BP-3 as a UV filter was 10% in Europe, which caused a single application of the cream to provide 4 g of BP-3 and resulted in a 52–61 mg/kg exposure to BP-3 for women (the average weight of European Union female residents is 65.8 kg, and the average US female resident weighs 76.4 kg). The BP-3 used for experiments was dissolved in peanut oil and subcutaneously injected in a volume of 10 ml/kg body weight. Control pregnant mice were injected with an equal volume of the solvent. In this study, 6 pregnant mice were injected with peanut oil only, and another 6 mice were treated with BP-3 (50 mg/kg). All procedures were performed in accordance with the National Institutes of Health Guidelines for the Care and Use of Laboratory Animals and the European Communities Council Directive for the Care and Use of Laboratory Animals (86/609/EEC) and were approved by the Committee for Laboratory Animal Welfare and the Ethics Committee of the Institute of Pharmacology PAS in Krakow, Poland (resolution nos. 1155/2015 and 489/2015). Animal care followed official governmental guidelines, and all efforts were made to minimize suffering as well as the number of animals used.

### Primary Neocortical Cell Cultures

Neocortical tissue for primary cultures was prepared from Swiss mouse embryos, which were exposed to BP-3 or peanut oil between 7 and 16 days of gestation. Afterwards, embryonic offspring were subjected to the isolation of cerebral tissue at 17 days of gestation. As described previously, the neocortical cells were suspended in estrogen-free neurobasal medium supplemented with B27 on poly-ornithine (0.01 mg/ml)-coated multiwell plates at a density of 2.0 × 10^5^ cells/cm^2^ and maintained at 37 °C in a humidified atmosphere containing 5% CO_2_ for 7 days in vitro (DIV) prior to experimentation. The number of astrocytes, as determined by the content of intermediate filament glial fibrillary acidic protein (GFAP), did not exceed 10% for all cultures as previously described [25, 28–30].

#### Observation

During the procedure of brain tissue isolation from embryos prenatally exposed to BP-3, oil droplets on the surface of isolated brain structures and in the isolation buffer were noticed.

### qPCR Analysis of Autophagy-Related Genes, Nuclear Receptors, and miRNAs

Total RNA was purified from 7 DIV neocortical cells using the RNeasy Mini Kit or the miRNeasy Mini Kit (Qiagen, Valencia, CA) according to the manufacturer’s instructions as previously described [25, 28–30]. The RNA quantification was spectrophotometrically determined at 260 nm and 260/280 nm (ND/1000 UV/Vis; Thermo Fisher NanoDrop, USA). cDNA was synthesized using the High Capacity cDNA–Reverse Transcription Kit (Thermo Fisher Scientific, USA) or the miScript II RT Kit (Qiagen, Valencia, CA). Both the reverse transcription reaction and qPCR were performed on a CFX96 Real-Time System (Bio-Rad, Hercules, CA, USA). The products of the reverse transcription reaction were amplified using TaqMan Gene Expression Master Mix containing TaqMan primer probes specific to the genes encoding *Hprt*, *Becn1*, *Nup62*, *Atg7*, *Map1lc3a*, *Map1lc3b*, *Rxrα*, *Rxrβ*, *Rxrγ*, *Pparγ*, *SNORD95*, *miR-19b*, *miR-33*, *miR-489*, *and miR-509.* Amplification was performed in a total volume of 20 μl containing 10 μl of TaqMan Gene Expression Master Mix and 1.0 μl of reverse transcription product as the PCR template. A standard qPCR procedure was utilized: 2 min at 50 °C and 10 min at 95 °C followed by 40 cycles of 15 s at 95 °C and 1 min at 60 °C. The threshold value (*C*_t_) for each sample was set during the exponential phase, and the delta *C*_t_ method was used for data analysis. To evaluate the reference gene expression, RefFinder web-based comprehensive tool has been used. *Hprt* (the gene encoding hypoxanthine phosphoribosyltransferase) was selected to use as a reference gene against *Becn1*, *Nup62*, *Atg7*, *Map1lc3a*, *Map1lc3b*, *Rxrα*, *Rxrβ*, *Rxrγ*, and *Pparγ*. *SNORD95* (the small nucleolar RNA, C/D box 95) has been chosen to be a reference gene in the cases of *miR-19b*, *miR-33*, *miR-489*, and *miR-509*. The results were obtained from three independent experiments.

### The ELISA Analyses of Autophagy-Related Factors, RXRs, and PPARγ

Briefly, the levels of BECLIN-1, NUP62, MAP1LC3A, MAP1LC3B, RXRα, RXRβ, RXRγ, and PPARγ in neocortical cells were detected with the use of ELISA assays, according to the manufacturer’s instructions [25, 28–30]. The standards and denaturated cell extracts were added to the precoated with monoclonal antibodies 96-well plate (specific for BECLIN-1, NUP62, MAP1LC3A, MAP1LC3B, RXRα, RXRβ, RXRγ, and PPARγ). After washing, the substrate solution was added to the wells. The enzymatic reaction yielded a blue product. The absorbance was measured at 450 nm and was proportional to the amount of specific proteins in the sample. The absorbance measurements were performed on Infinite M200pro microplate reader (Tecan, Männedorf, Zürich, Switzerland). The protein concentration of each sample was determined using Bradford reagent (Bio-Rad Protein Assay). The protein levels of the experimental samples are expressed as a percentage of control ± SE and in pg/μg protein. The results were obtained from three independent experiments.

### Western Blot Analyses

To visualize specific proteins, western blot analyses have been used. Firstly, the cells were lysed in RIPA lysis buffer with protease inhibitor cocktail. The solution has been sonicated and centrifuged at 15,000×*g* for 20 min at 4 °C. The protein concentrations were determined using Bradford reagent (Bio-Rad Protein Assay) with bovine serum albumin (BSA) as the standard, and next the samples (40 μg of total protein) were reconstituted and denaturated in the adequate amount of Laemmli sample buffer. Subsequently, samples were separated on Mini-PROTEAN TGX Precast Gels using a Bio-Rad Mini-Protean II Electrophoresis Cell as previously described [25, 28–30]. After electrophoretic separation, the proteins were transferred from gel to PVDF membranes (Millipore, Bedford, MA, USA) using the Bio-Rad Mini Trans-Blot apparatus (Bio-Rad, Hercules, CA, USA). Afterwards, the membranes were washed, and the non-specific binding sites were blocked with 5% dried milk and 0.2% Tween-20 in 0.02 M TBS (Tris-buffered saline) for 2 h with shaking. This was followed by overnight incubation (at 4 °C) with one of the following primary antibodies diluted in TBS/Tween: anti-BECLIN-1 rabbit polyclonal antibody (diluted 1:100), anti-NUP62 mouse rabbit polyclonal antibody (diluted 1:100), anti-MAP1LC3A/B mouse monoclonal antibody (diluted 1:100), anti-ATG7 rabbit monoclonal antibody (diluted 1:1000), rabbit polyclonal anti-RXRα antibody (1:100), mouse monoclonal anti-RXRβ antibody (1:100), mouse monoclonal anti-RXRγ antibody (1:100), anti-PPARγ mouse polyclonal antibody (diluted 1:100), or anti-β-ACTIN mouse monoclonal antibody (diluted 1:3000). The signals were developed by chemiluminescence (ECL) using BM Chemiluminescence Blotting Substrate (Roche Diagnostics GmBH) and visualized using a Luminescent Image Analyzer Fuji-Las 4000 (Fuji, Japan). Immunoreactive bands were quantified using an image analyzer (MultiGauge V3.0). Protein levels of the samples were obtained from three independent experiments.

### Profiling of miRNAs Using Microarray Assays

The miRNeasy Mini Kit was used for RNA purification (including RNA from approx. 18 nucleotides) using spin columns to extract RNA from neocortical cells that were cultured for 7 DIV. The entire procedure was conducted according to the manufacturer’s protocol. The concentration of RNA was determined by measuring the absorbance at 260 nm and 260/280 nm (ND/1000 UV/Vis; Thermo Fisher NanoDrop, USA). Total RNA containing miRNA was required for miScript miRNA PCR Arrays (Qiagen, CA, USA). The reverse transcription reaction was conducted using miScript II RT Kit (Qiagen, CA, USA) according to the manufacturer’s instruction. Thus, the material obtained enabled qPCR profiling of mature miRNA using miScript miRNA PCR Arrays (Qiagen, CA, USA). The *C*_t_ values for all wells were exported to a blank Excel spreadsheet and were analyzed with web-based software (pcrdataanalysis.sabiosciences.com/mirna). To normalize miRNA expression, the sn/snoRNA was used (*SNORD61*, *SNORD68 SNORD95*, *SNORD96A*). The results were obtained from two independent experiments. The reverse transcription reaction and qPCR profiling were performed on a CFX96 Real-Time System (Bio-Rad, Hercules, CA, USA).

### Pathway-Focused Gene Expression Analysis Using Microarray Assays

RT^2^ Profiler PCR Arrays is a qPCR-based analysis for multiple gene expression profiling. The total RNA was extracted from neocortical cells cultured for 7 DIV using the RNeasy Mini Kit (Qiagen, CA, USA) according to the manufacturer’s instructions and spectrophotometrically measured. A total of 1 μg of RNA was reverse-transcribed to cDNA using the RT^2^ First Strand Kit (Qiagen, CA, USA) and suspended in a final volume of 20 μl as previously described [25, 29, 30]. Each cDNA sample was prepared for further use in qPCR with RT^2^ SYBR Green Mastermix. To analyze the signaling pathway, the RT^2^ Profiler™ PCR Array System (Qiagen, CA, USA) was used according to the manufacturer’s protocol. The *C*_t_ values for all wells were exported to a blank Excel spreadsheet and were analyzed with a web-based software (www.SABiosciences.com/pcrarraydataanalysis.php). *Actb* (β-actin) and *Gapdh* (glyceraldehyde-3-phosphate dehydrogenase) were used as reference genes. The results were obtained from two independent experiments.

### Measurement of Permeability Through the Blood-Brain Barrier

The BBB Kit™ (PharmaCo-Cell Company Ltd., Nagasaki, Japan) was used for the evaluation of BP-3 permeability through the BBB. The BBB Kit™ (RBT-24) is a new in vitro model of the BBB that consists of primary culture of rat (Wistar rat) brain capillary endothelial cells, pericytes, and astrocytes. The whole procedure was conducted according to the manufacturer’s protocol. In brief, BP-3 was added to the upper (luminal, blood-side) insert. After 30 min, the samples were collected from the lower (abluminal, brain-side) compartment. Subsequently, the BP-3 concentrations were measured. To each sample of 900 μl of abluminal compartment solution, 300 μl of brine and 500 μl of ethyl acetate were added. Then, vials were stirred on a vortex, and the phases were allowed to separate. The concentration of BP-3 in the organic phase was evaluated by measuring the total ion current (TIC) for the molecular mass of BP-3 on a TQD Waters mass spectrometer with ESI+ ionization, coupled with an H-class UPLC. Each sample was measured in triplicate. The samples were separated on an ACQUITY UPLC BEH C18 1.7 μm 2.1 × 50 mm column, using a 4-min gradient (0.3 ml/min) increasing from 80% H_2_O–20% ACN to 100% ACN at 2.5 min then 100% ACN for 0.5 min and decreasing back to 80% H_2_O–20% ACN at 4 min. The BP-3 was eluted at 3.3 min. The parameters of ionization were as follows: cone voltage 30 V, capillary voltage 3.95 kV, extractor voltage 2.2 V, RF 0.1 V, source temperature 150 °C, desolvation temperature 250 °C. The number of replicates was 6. The apparent permeability coefficient (*P*_app_) was calculated as described below:

$$ P\mathrm{app}\ \left(\frac{\mathrm{cm}}{\min}\right)=\frac{Va}{A\times \left[C\right]\mathrm{luminal}}\times \frac{\Delta \ \left[C\right]\mathrm{abluminal}}{\Delta  t} $$

where *A* is the culture area (cm^2^); *V*_a_ is the volume of assay buffer in the luminal side; Δ[*C*] abluminal is the concentration of sample in the abluminal side; [*C*] luminal is the initial concentration of sample added into the luminal side; and Δ*t* is the assay period (min).

### Detection of Autophagosome Formation

Neocortical cells on 96-well plates were used to detect the autophagosome formation according to the manufacturer’s instruction for the ELISA-based format—Autophagy Assay Kit as previously described [29]. Measurement of the autophagy in the neocortical cells was performed using a proprietary fluorescent autophagosome marker (*λ*_ex_ = 333 nm/*λ*_em_ = 518 nm). The autophagosomes were detected using an Infinite M200pro microplate reader (Tecan, Männedorf, Zürich, Switzerland).

### Measurement of HDAC and HAT Activity

The Histone Deacetylase (HDAC) Assay Kit and the Histone Acetyltransferase (HAT) Activity Fluorometric Assay Kit (Sigma-Aldrich, St. Louis, MO, USA) were used to detect enzyme activity. The procedures were performed according to the manufacturer’s protocol as previously described [29]. Regarding the HDAC kit, the measured fluorescence at *λ*_ex_ = 365 nm/*λ*_em_ = 460 nm was proportional to the deacetylation activity. In the HAT assay, the generated product of histone acetyltransferase activity was detected fluorometrically at *λ*_ex_ = 535 nm/*λ*_em_ = 587 nm. The HAT kit included an active nuclear extract to be used as a positive control and in the HDAC assay contained HeLa cell lysate as a positive control. The abovementioned assays provided positive and negative controls, as well as all the reagents required for analysis. Measurements were performed using an Infinite M200 pro microplate reader (Tecan, Männedorf, Zürich, Switzerland).

### Measurement of Sirtuin Activity

The sirtuin activity was measured using the Sirtuin Activity Assay Kit (BioVision, CA, USA) according to the manufacturer’s instructions. The acetylated p53-AFC substrate was deacetylated by sirtuins in the presence of NAD^+^. The developer provided in the assay cleaved the deacetylated p53-AFC substrate and released the fluorescent group, which was detected fluorometrically at *λ*_ex_ = 400 nm/*λ*_em_ = 505 nm. Since HDAC are also able to deacetylate p53-AFC substrate, trichostatin A was added to the reaction to specifically inhibit HDAC activity in the samples. The fluorescence was detected using an Infinite M200pro microplate reader (Tecan, Männedorf, Zürich, Switzerland).

### Measurement of Global Protein Sumoylation

The Global Protein Sumoylation Assay Kit (Abcam, Cambridge, UK) was performed to quantify sumoylated protein levels in the samples. The whole procedure was conducted according to the manufacturer’s protocol. The assay provided all necessary reagents to detect sumoylated protein with an anti-SUMO antibody. The kit included positive control and thus allowed the quantification of protein sumoylation. The absorbance was measured at 450 nm using an Infinite M200pro microplate reader (Tecan, Männedorf, Zürich, Switzerland).

### Data Analysis

Statistical tests were performed on raw data that were expressed as the mean arbitrary absorbance or as the fluorescence units per well containing 50,000 cells (measurements of autophagosome formation); the fluorescence units per 1.5 million cells (qPCR, microarray RT^2^ Profiler™ PCR, HDAC, and sirtuin activities); the absorbance units per 1.5 million cells (HAT activity and global protein sumoylation), the mean optical density per 40 μg of protein (western blotting); or pg of BECLIN-1, NUP62, MAP1LC3A, MAP1LC3B, RXRα, RXRβ, RXRγ, and PPARγ per μg of total protein (ELISA). One-way analysis of variance (ANOVA) was preceded by Levene’s test of homogeneity of variances and was used to determine overall significance. Differences between the control and experimental groups were assessed using a post hoc Newman–Keuls test, and significant differences were designated *p < 0.05, **p < 0.01, and ***p < 0.001 versus control cultures. The results were expressed as the mean ± SE of two to three independent experiments. The number of replicates in each experiment ranged from 2 to 3, except for the measurements of autophagosomes formation, which contained 8 replicates and BBB permeability with 6 replicates.

## Results

### Effects of Prenatal Exposure to BP-3 on the Expression of Autophagy-Related Genes and Autophagosomes Formation

**Fig. 1:**
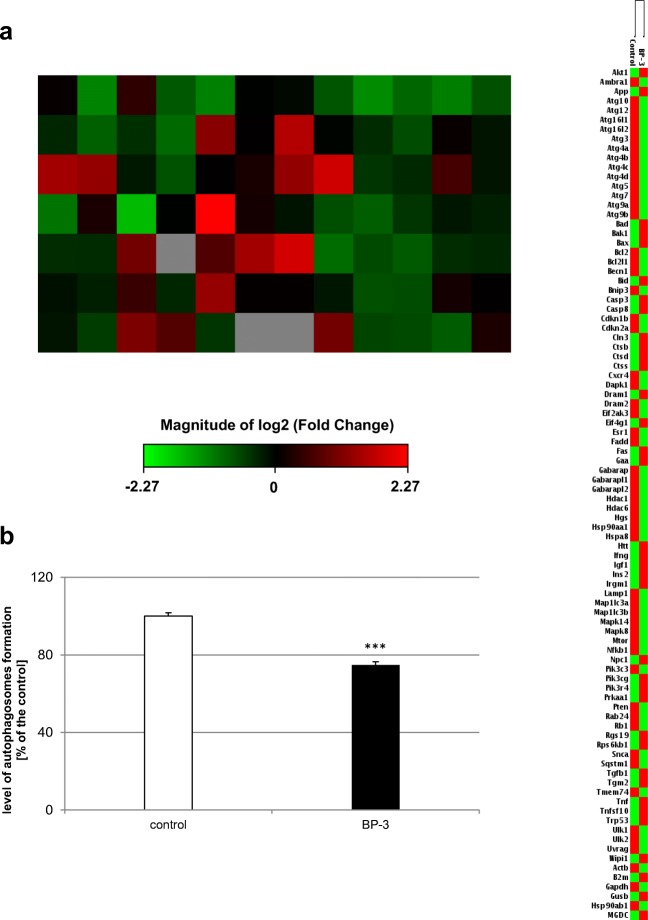
Prenatal BP-3 exposure decreased expression of autophagy-related genes and autophagosomes formation in embryonic neuronal cells. Analysis with microarray demonstrated 38 genes that were significantly differentially expressed in the BP-3-treated group in comparison to the control groups. Among them, 22 genes were downregulated (green color) and 16 genes were upregulated (red color) in the BP-3-treated samples. Each value represents the mean of two independent experiments (**a**). Prenatal BP-3 decreased the formation of autophagosomes in mouse embryonic neuronal cells. The data are expressed as the mean ± SE of three independent experiments, consisting of eight replicates per group. ***p < 0.001 versus control cultures (**b**)

In neocortical cultures derived from embryos that were prenatally exposed to BP-3, the analysis of the expression profiles of 84 genes involved in autophagy revealed that 38 genes were differentially expressed in neocortical cells from BP-3-exposed embryos than in the neocortical cells from the control (peanut oil-treated) embryos. Among them, 22 genes were downregulated (green color), and 16 genes were upregulated (red color) in the BP-3-treated samples. The downregulated genes were *Ambra1*, *Atg10*, *Atg12*, *Atg3*, *Atg4a*, *Atg4b*, *Atg4c*, *Atg4d*, *Atg7*, *Atg9b*, *Becn1*, *Cdkn2a*, *Eif2ak3*, *Esr1*, *Gabarapl1*, *Gabarapl2*, *Lamp1*, *Map1lc3a*, *Map1lc3b*, *Ulk1*, *Ulk2*, and *Uvrag*. The upregulated genes were *Bad*, *Bax*, *Casp3*, *Casp8*, *Ctsd*, *Ctss*, *Dram1*, *Fas*, *Htt*, *Igf1*, *Ins2*, *Irgm1*, *Pik3cg*, *Tgfb1*, *Tgm2*, and *Trp53* (Fig. 1a).

Treatment of pregnant mice with BP-3 reduced the level of autophagosomes in embryonic neuronal cells by 26% compared to the control value (Fig. 1b).

### Effects of Prenatally Administered BP-3 on the mRNA and Protein Expression Levels of Autophagy-Related Factors

**Fig. 2:**
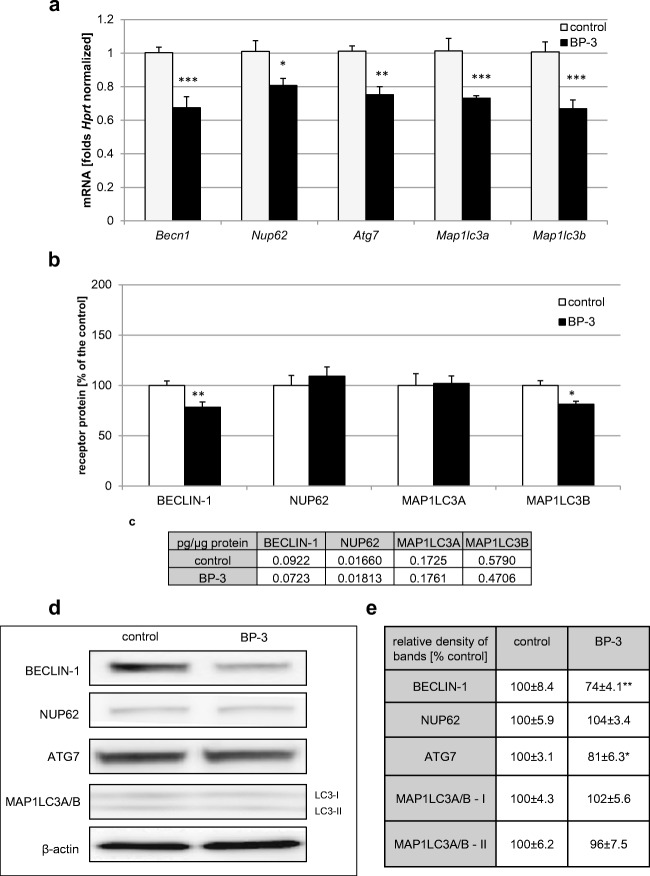
Prenatal BP-3 diminished the expression of autophagy-related factors in mouse embryonic neurons. The effect of prenatal BP-3 on the mRNA expression levels of *Becn1*, *Nup62*, *Atg7*, *Map1lc3a*, and *Map1lc3b* (**a**) in neocortical cultures. **b**–**e** The effects of prenatally administered BP-3 on the protein levels of autophagy-related factors (BECLIN-1, NUP62, MAP1LC3A, and MAP1LC3B). The concentrations of the protein were measured using specific ELISA and are presented as a percentage of the control (**b**) and as pg of specific protein per μg of total protein (**c**). For the western blot analyses, protein samples were denatured, electrophoretically separated, transferred to PVDF membranes, and subjected to immunolabeling (**d**). The relative protein levels of BECLIN-1, NUP62, ATG 7, MAP1LC3A, and MAP1LC3B were presented as a percentage of the control (**e**). Each bar represents the mean of three independent experiments ± SE. The number of replicates in each experiment ranged from 2 to 3. *p < 0.05, **p < 0.01, and ***p < 0.001 versus control cultures

According to our study, prenatal administration of BP-3 impaired the mRNA levels of autophagy-related factors in embryonic neurons. Exposure of the offspring to BP-3 caused a decrease in *Becn1*, *Nup62*, *Atg7*, *Map1lc3a*, and *Map1lc3b* mRNA by 20–34% (Fig. 2a). ELISA kits revealed that embryos exposed to BP-3 expressed decreased levels of BECLIN-1 protein (0.0723 pg/μg of total protein; 78% of the control) and MAP1LC3B protein (0.47 pg/μg of total protein; 81% of the control) in neocortical cells, but no effect on the levels of NUP62 and MAP1LC3A proteins was noticed (Fig. 2b, c). Western blot analysis indicated that prenatal exposure to BP-3 decreased the relative BECLIN1 and ATG7 protein levels by 26% and 19%, respectively. No changes were observed with respect to the relative protein levels of NUP62, MAP1LC3A, and MAP1LC3B (Fig. 2d, e).

In our study, BP-3 evoked a decrease in mRNA expression of all the abovementioned autophagy-related factors that was paralleled by a decrease in the protein levels of BECLIN-1, ATG7, and MAP1LC3B. The protein levels of the other factors remained unchanged. ELISA unraveled the BP-3-evoked attenuation of MAP1LC3B, but it was not supported by western blot analysis.

### Effects of Prenatal Exposure to BP-3 on the mRNA and Protein Expression Levels of Retinoid X Receptors (RXRα, RXRβ, RXRγ) and PPARγ

**Fig. 3:**
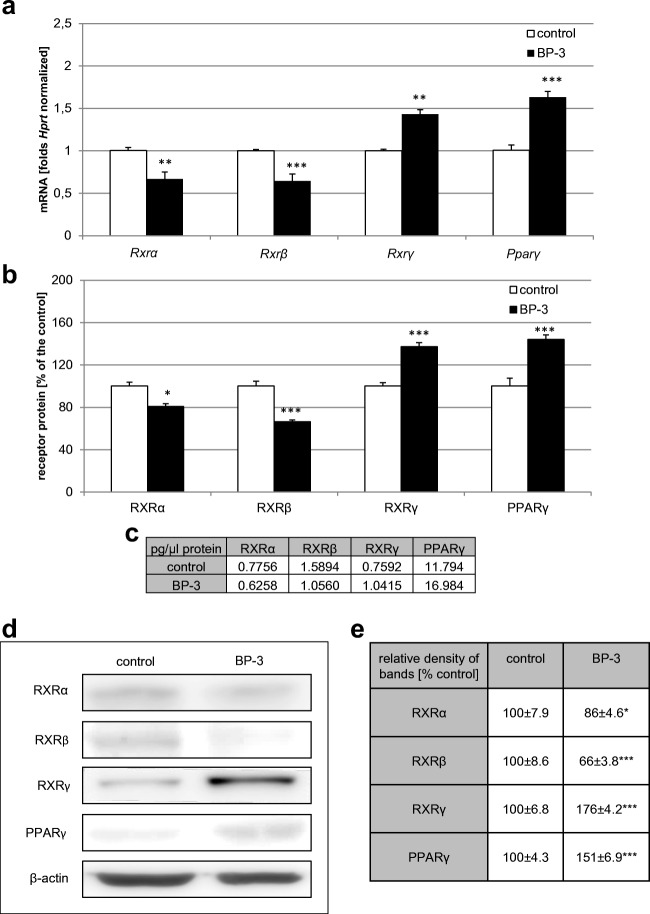
Prenatal BP-3 altered the expression of RXRα, RXRβ, RXRγ, and PPARγ in mouse embryonic neurons. The effect of prenatal BP-3 on the mRNA expression levels of *Rxrα*, *Rxrβ*, *Rxrγ*, and *Pparγ* (**a**) in neocortical cultures. **b**–**e** The effects of prenatally administered BP-3 on the protein levels of RXRα, RXRβ, RXRγ, and PPARγ. The concentrations of the proteins were measured using specific ELISA kits and are presented as a percentage of the control (**b**) and as pg of specific protein per μg of total protein (**c**). The western blot analyses were demonstrated as immunolabeling (**d**), and the relative protein levels of RXRα, RXRβ, RXRγ, and PPARγ were presented as a percentage of the control (**e**). Each bar or value represents the mean of three independent experiments ± SE and the number of replicates in each experiment ranged from 2 to 3. *p < 0.05, **p < 0.01, and ***p < 0.001 versus control cultures

Our data demonstrated that prenatal exposure to BP-3 altered the mRNA levels of *Rxrα*, *Rxrβ*, *Rxrγ*, and *Pparγ*. The exposure decreased levels of *Rxrα* and *Rxrβ* mRNA by 33% and 36%, respectively, but it increased levels of *Rxrγ* and *Pparγ* mRNA by 43% and 62%, respectively (Fig. 3a). ELISA kits revealed that prenatal treatment with BP-3 decreased levels of RXRα and RXRβ protein by 19 and 34% (equal to 0.62 and 1.06 pg/μg of total protein, respectively), whereas the RXRγ and PPARγ protein levels increased to 137% and 144% of the control value (equal to 1.04 and 17 pg/μg of total protein, respectively), Fig. 3b, c. Western blot analysis determined a decrease in the relative protein levels of RXRα and RXRβ of 14–34% and an increase in RXRγ and PPARγ protein levels of 76% and 51%, respectively in (Fig. 3d, e).

### Effects of Prenatal Exposure to BP-3 on the Expression of miRNAs

**Fig. 4:**
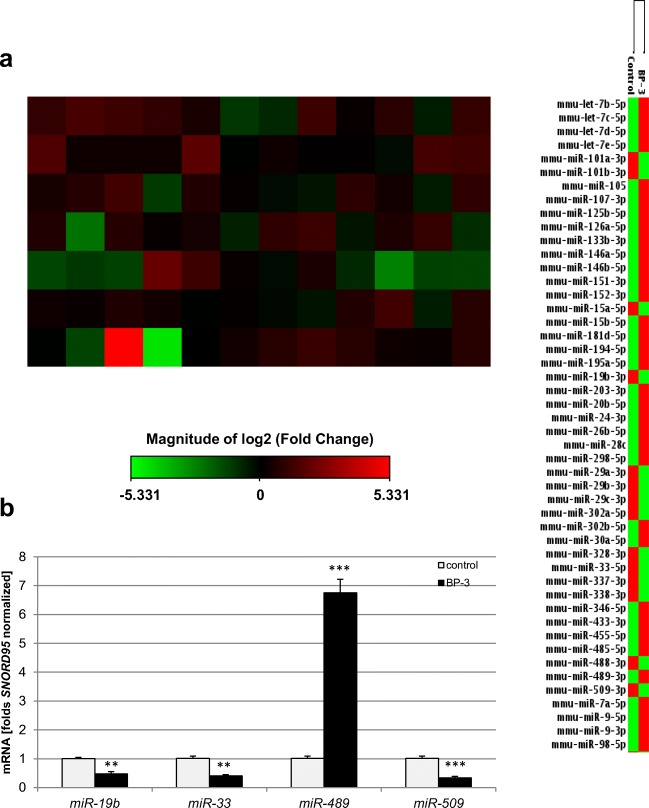
Prenatal BP-3 exposure modified the expression of miRNAs engaged in neuronal development or in the progression of neurological diseases in mouse embryonic neurons. Gene expression patterns of miRNAs showing that the expression of 36 genes was significantly different between the control and BP-3-treated groups. Among altered miRNAs, 23 genes were upregulated (red color), and 13 genes were downregulated (green color) in the BP-3-treated samples compared to the controls (**a**). Each bar represents the mean of two independent experiments ± SE. The qPCR technique was employed to validate the expression levels of the most dysregulated miRNAs from the microarray assay (**b**). Each bar represents the mean of three independent experiments and the number of experimental replicates ranged from 2 to 3 ± SE. **p < 0.01 and ***p < 0.001 versus control cultures

The use of the qPCR microarray technique allowed the analysis of the expression profiles of 84 miRNA that are involved during either neuronal development or the progression of neurological diseases. After prenatal exposure to BP-3, these data showed that 36 miRNAs genes were differentially expressed in neuronal cells from BP-3-exposed embryos compared to the neocortical cells from the control peanut oil-treated embryos. Among these genes, 23 were upregulated (red color), and 13 were downregulated (green color). The upregulated genes were *mmu-let-7b-5p*, *mmu-let-7c-5p*, *mmu-let-7d-5p*, *mmu-let-7e-5p*, *mmu-miR-105*, *mmu-miR-107-3p*, *mmu-miR-125b-5p*, *mmu-miR-126a-5p*, *mmu-miR-133b-3p*, *mmu-miR-146a-5p*, *mmu-miR-146b-5p*, *mmu-miR-152-3p*, *mmu-miR-181d-5p*, *mmu-miR-194-5p*, *mmu-miR-24-3p*, *mmu-miR-26b-5p*, *mmu-miR-298-5p*, *mmu-miR-302b-5p*, *mmu-miR-30a-5p*, *mmu-miR-455-5p*, *mmu-miR-489-3p*, *mmu-miR-7a-5p*, and *mmu-miR-9-5p.* The downregulated genes were *mmu-miR-101a-3p*, *mmu-miR-15a-5p*, *mmu-miR-19b-3p*, *mmu-miR-22-3p*, *mmu-miR-29a-3p*, *mmu-miR-29b-3p*, *mmu-miR-29c-3p*, *mmu-miR-302a-5p*, *mmu-miR-33-5p*, *mmu-miR-337-3p*, *mmu-miR-338-3p*, *mmu-miR-488-3p*, and *mmu-miR-509-3p* (Fig. 4a).

miRNAs differentially expressed in our present study have been linked to autistic disorder (*miR-146b-5p*, *miR-15a-5p*, *miR-181d-5p*, and *miR-7a-5p*), schizophrenia (*let-7d-5p*, *let-7e-5p*, *miR-105*, *miR-107-3p*, *miR-126-5p*, *miR-152-3p*, *miR-15a-5p*, *miR-15b-5p*, *miR-195a-5p*, *miR-20b-5p*, *miR-24-3p*, *miR-26b-5p*, *miR-29a-3p*, *miR-29b-3p*, *miR-29c-3p*, *miR-302a-5p*, *miR-302b-5p*, *miR-30a-5p*, *miR-33-5p*, *miR-346-5p*, *miR-455-5p*, *miR-489-3p*, *miR-7a-5p*, and *miR-9-3p*), anxiety disorder (*miR-485-5p* and *miR-509-3p*), Tourette’s syndrome (*miR-24-3p*), Alzheimer’s disease (AD) (*let-7b-5p*, *let-7c-5p*, *let-7d-5p*, *let-7e-5p*, *miR-101b-3p*, *miR-107-3p*, *miR-146a-5p*, *miR-151-3p*, *miR-15a-5p*, *miR-15b-5p*, *miR-194-5p*, *miR-19b-3p*, *miR-24-3p*, *miR-26b-5p*, *miR-28a-5p*, *miR-298-5p*, *miR-29a-3p*, *miR-29b-3p*, *miR-29c-3p*, *miR-328-3p*, *miR-346-5p*, *miR-433-3p*, *miR-488-3p*, *miR-9-5p*, and *miR-98-5p)*, prion disease (*let-7b-5p*, *miR-146a-5p*, *miR-203-3p*, *miR-337-3p*, and *miR-338-3p*), Huntington’s disease (HD) (*miR-29a-3p*, *miR-29b-3p*, *miR-9-5p*, and *miR-9-3p*), Parkinson’s disease (PD) (*miR-133b-3p*, *miR-433-3p*, and *miR-7a-5p*), and spinocerebellar ataxia 1 (*miR-101a-3p* and *miR-19b-3p*).

The qPCR technique was employed to validate the expression levels of the most dysregulated miRNAs (at least 2-fold change) from the microarray assay, i.e., *miR-19b*, *miR-33*, *miR-489* and *miR-509*. This qPCR analysis confirmed that *miR-19b*, *miR-33* and *miR-509* mRNAs were significantly reduced by 52–68%, whereas *miR-489* mRNA was extremely elevated to 675% of the control value (Fig. 4b).

### Effects of Prenatal Exposure to BP-3 on HAT, HDAC, and Sirtuin Activities as well as Global Protein Sumoylation

**Fig. 5:**
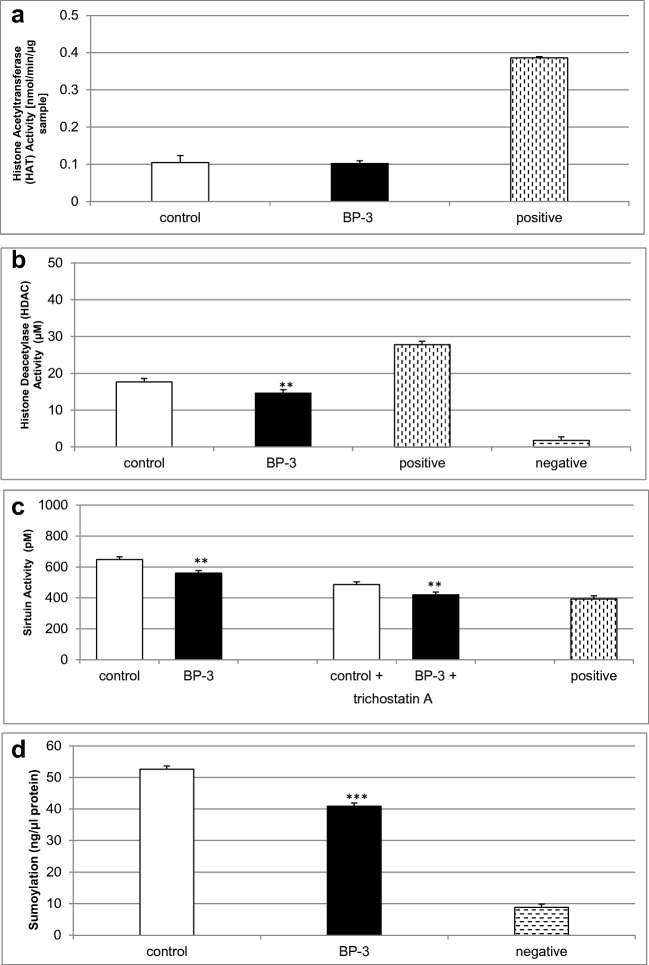
Reduced HDAC and sirtuin activities as well as decreased sumoylation in mouse neurons prenatally treated with BP-3. The influence of prenatal BP-3 on HAT (**a**), HDAC (**b**), and sirtuin (**c**) activities as well as global protein sumoylation (**d**) in cortical cultures at 7 DIV. Each bar represents the mean of three independent experiments ± SE. The number of replicates in each experiment ranged from 2 to 3. **p < 0.01 and ***p < 0.001 versus control cultures

Prenatal exposure to BP-3 reduced HDAC and sirtuin activities to 14 μM (i.e., 82% of the control) and 560 pM (i.e., 86% of the control), respectively (Fig. 5b, c). In mouse embryonic neurons prenatally treated with BP-3, a decrease in global protein sumoylation to 41 ng/μl (i.e., 78% of the control value) was observed (Fig. 5d). BP-3 administered during pregnancy did not affect HAT activity in the neocortical neurons of embryonic offspring (Fig. 5a).

### Influence of Prenatally Administered BP-3 on the Expression of Neurogenesis-Related Genes and Neurotransmitter Receptor Genes

**Fig. 6:**
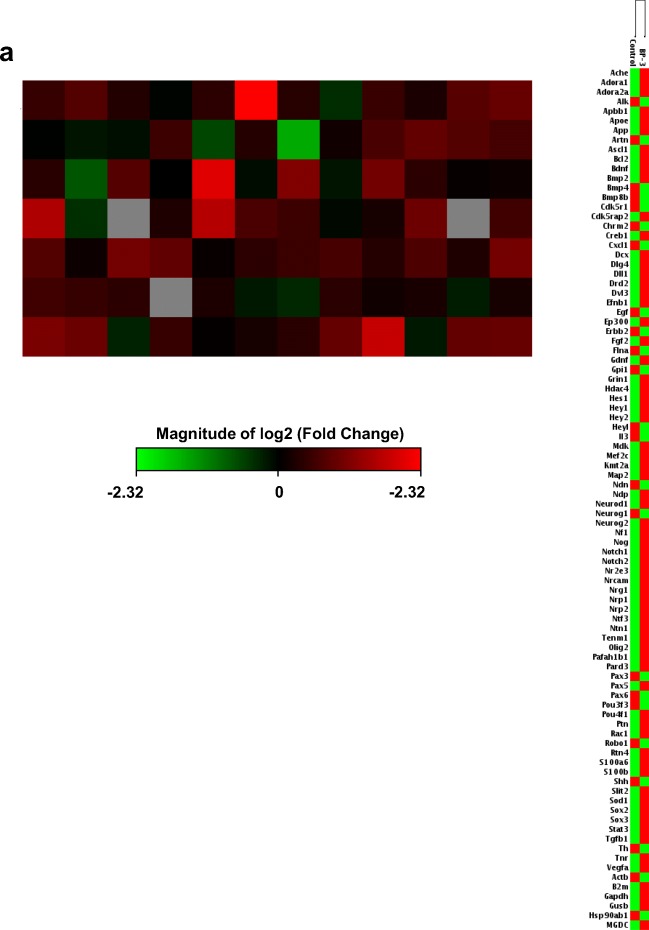

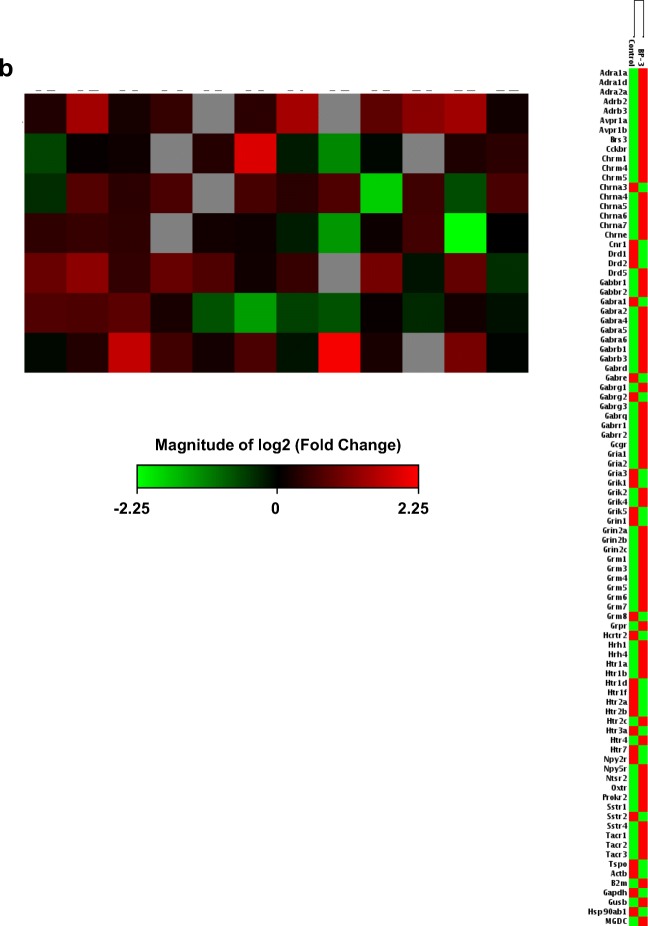
Prenatal BP-3 exposure modified the expression of neurogenesis-related and neurotransmitter receptor genes in mouse embryonic neurons. Gene expression patterns of neurogenesis demonstrated the 24 genes that were significantly differentially expressed between the control and BP-3-treated groups. Among these genes, 22 of them were upregulated (red color), and 2 genes were downregulated (green color) in the BP-3-treated samples compared to the control (**a**). Neurotransmitter receptor gene expression displayed 23 genes significantly differentially expressed between the control and BP-3-treated groups; 16 genes were upregulated (red color), and 7 genes were downregulated (green color) in the BP-3-treated samples compared to the controls (**b**)

To support our data related to autophagy and epigenetic status in response to prenatal exposure to BP-3, we analyzed the expression profiles of 84 neurogenesis-related genes and 84 neurotransmitter receptor genes. The data showed that 24 genes involved in neurogenesis were differentially expressed in neocortical cells from BP-3-exposed embryos, of which 22 were upregulated (red color) and 2 were downregulated (green color). The upregulated genes related to neurogenesis were *Adora1*, *Apoe*, *Bdnf*, *Bmp2*, *Dll1*, *Ep300*, *Fgf2*, *Gdnf*, *Grin1*, *Hey2*, *Mef2c*, *Neurod1*, *Nf1*, *Notch1*, *Notch2*, *Tenm1*, *S100a6*, *S100b*, *Stat3*, *Tgfb1*, *Tnr*, and *Vegfa*, while the downregulated genes were *Cxcl1* and *Egf* (Fig. 6a). Moreover, in BP-3-treated embryos, 23 neurotransmitter receptor genes were differentially expressed. Among them, 16 genes were upregulated, i.e., *Adra1d*, *Avpr1b*, *Cckbr*, *Chrm1*, *Chrm4*, *Chrne*, *Grin2a*, *Grin2b*, *Grm1*, *Grm7*, *Grpr*, *Hrh1*, *Htr1a*, *Ntsr2*, *Sstr4*, and *Tacr3*, and 7 genes were downregulated: *Drd1*, *Gabre*, *Grik1*, *Grik5*, *Htr1d*, *Htr1f*, and *Htr2b* (Fig. 6b).

### Measurement of BP-3 Permeability Value Using the BBB Kit™

The evaluation of BP-3 (25 μM) permeability with the use of the BBB Kit™ (RBT-24) revealed that BP-3 is able to cross the BBB. The permeability assay, which calculates a *P*_app_ value (× 10^−6^ cm/s) for tested compounds, showed that BP-3 has mean value of 10 (Table 1). This value positioned BP-3 with a good permeability coefficient, since compounds with *P*_app_ < 2, 2–10, 10–20, and > 20 can be classified as having a very low, low, good, and very good permeability capacity, respectively [31].

**Table 1:** BP-3 permeability through the BBB. BP-3 (25 μM) has a good permeability coefficient through the BBB. Evaluation of the permeability properties was measured according to the *P*_app_ value. The concentration of BP-3 in the organic phase was evaluated by measuring the total ion current (TIC) for the molecular mass of BP-3 on a TQD Waters mass spectrometer with ESI+ ionization, coupled with an H-class UPLC. The number of replicates was 6

Replicate	*P*app (cm/min)	*P*app (10–6) cm/s
1	0.000588111	9.803137744
2	0.00052516	8.753816746
3	0.000544321	9.073217155
4	0.000814718	13.58043162
5	0.000557757	9.29717601
6	0.000600898	10.01629517
**Mean ± SE**	0.00061 ± 0.000043	**10.10 ± 0.7238**

## Discussion

Our research presented here revealed for the first time that prenatal exposure to BP-3 impairs autophagy, disrupts the levels of retinoid X and peroxisome proliferator-activated receptors, alters epigenetic status (i.e., attenuates HDAC and sirtuin activities), inhibits post-transcriptional modifications in terms of global sumoylation, and dysregulates expression of neurogenesis- and neurotransmitter-related genes and specific miRNAs involved in developmental and degenerative pathologies of the nervous system.

Considering current population studies, there is no doubt that BP-3 easily reaches the fetus and affects its development. Indeed, based on our research, BP-3 is able to cross the BBB (with a good permeability factor), which may directly influence the developing brain, conditioning it for cerebral damage. Our previous study demonstrated that prenatal exposure to BP-3 causes severe apoptosis and neurotoxicity, evokes global DNA hypomethylation, alters methylation status of apoptosis-related and estrogen receptors genes, and disrupts estrogen receptors expression [25]. Taking these data into account, we strongly suggest that BP-3 can significantly affect the neural development, which may be the fetal basis of the adult onset of nervous system disease.

In the present study, administration of an environmentally relevant dose of BP-3 (50 mg/kg) to pregnant mice evoked significant autophagy inhibition in neocortical cells from their embryonic offspring. The impairment of autophagic processes has been confirmed by a decrease in autophagosomes formation and a significant downregulation of 22 autophagy-related genes measured by microarray analysis and validated by qPCR. Furthermore, autophagy inhibition has been detected by decreased levels of autophagy-involved proteins, i.e., BECLIN-1, ATG7, and MAP1LC3B. This is in line with our previous study, in which 25 μM BP-3 in vitro was able to reduce the autophagy process in neuronal cells via downregulating specific genes as well as reducing the MAP1LC3 ratio and diminishing autophagosome formation [29]. BECLIN-1 plays a critical role in autophagy induction and its lowered expression after prenatal BP-3 can result in a gross impairment of autophagy. Except for our data, there have been no studies showing the impact of BP-3 on the course of autophagy in mammalian neurons. However, another environmental chemical pollutant, tri-O-cresyl phosphate (TOCP), has been demonstrated to cause a decrease in BECLIN-1 expression, which was associated with significant dysfunction in the nervous system in hens [32, 33]. Taking into account that prenatal exposure to BP-3 caused substantial reduction of BECLIN-1, we suggest that BP-3 may increase the risk of schizophrenia since in brains of schizophrenia patients autophagy is impaired, particularly BECLIN-1 expression level is decreased by 40% [34–36]. Weakened autophagy has also been postulated to be involved in the origins of several neurodegenerative diseases, such as AD, PD, HD, amyotrophic lateral sclerosis (ALS) and multiple sclerosis (MS), being responsible for β-amyloid/tau, α-synuclein, or mHtt clearance [37].

In our study, in addition to inhibition of autophagy, prenatal exposure to BP-3 caused a decrease in the mRNA and protein expression levels of RXRα and RXRβ, whereas RXRγ and PPARγ showed an increased expression pattern. Our current data on the prenatal exposure of mouse brains to BP-3 confirm our previous observation based on in vitro treatment of mouse neurons with 25 μM BP-3 [29, 30]. Previously, we showed the importance of RXRs signaling in the propagation of dichlorodiphenyldichloroethylene (DDE) and nonylphenol apoptotic and neurotoxic effects [28, 38]. The RXRs, mainly RXRα, has been classified as a heterodimerization partner of other nuclear receptors. Thus, a deficiency of RXRα and RXRβ, as occurred in the current study, can have far-reaching consequences regarding the number of RXR-dependent dimerization partners (such as nuclear receptor-related 1 protein (Nurr1), nerve growth factor IB (Nur77 or NR4A1), retinoid acid receptor (RAR), and PPARs) involved in the coordination of neurogenesis, neuronal cell differentiation, and lipid signaling [39–42]. A decrease in the level of RXRβ has already been correlated with schizophrenia, and RXR agonist—bexarotene—is currently in the third clinical trial phase for the reduction of positive symptoms of schizophrenia [22, 43–45]. The RXRγ signaling pathway has been implicated in modulation of despair behaviors and working memory, controlling the affective behaviors by regulation of dopaminergic signaling and accelerating the CNS remyelination [20, 46, 47]. Furthermore, PPARγ has been demonstrated to be involved in cerebral development and peripheral nervous system myelination [48, 49]. In addition, activation of RXRs or/and PPARγ by receptor agonists has been associated with neuroprotection from several diseases such as AD, PD, ALS, MS, and stroke [50–57]. Although, in our study, BP-3 stimulated PPARγ expression, it inhibited expression of PPARγ heterodimerization partner, i.e., RXRα that suggests an impairment of PPARγ neuroprotective capacity in response to BP-3. Previously, we showed that prenatally administered BP-3 (50 mg/kg) induced apoptosis and caused neurotoxicity that was accompanied by impaired ESR1/ESR2 expression, enhanced GPER1, and altered methylation status in the mouse neuronal cells [25]. Taking into account our previous and present data, we hypothesize that prenatal exposure to BP-3 may be linked to developmental abnormalities and the etiology of neural degeneration.

In the present study, this hypothesis has been partially confirmed by the microarray analyses of neurogenesis- and neurotransmitter-related genes. These involve changed expression of brain-derived neurotrophic factor (BDNF), epidermal growth factor (EGF), glial cell-derived neurotrophic factor (GDNF), Notch, myocyte-specific enhancer factor 2C (MEF2C), and apoE as well as altered levels of adrenergic, cholinergic (e.g., cholinergic receptor nicotinic alpha 4 subunit (CHRNA4)), dopaminergic, GABA-ergic, glutamatergic (e.g., glutamate ionotropic receptor NMDA type subunit 2A (GRIN2A)), and serotoninergic receptors. In this study, we demonstrated that prenatal exposure to BP-3 caused substantial increase in *Mef2c*, *Grin2a*, and *Chrna4* which corresponds to upregulation of these genes in brains of schizophrenia patients as detected in post-mortem [58, 59]. In the present study, during the procedure of brain tissue isolation from embryos prenatally exposed to BP-3, oil droplets on the surface of isolated brain structures and in the isolation buffer were noticed. These droplets may be due to inappropriate metabolism resulting from abnormal expression of nuclear receptors including RXRs and PPARγ. Lipid metabolism deficiency resulting from the downregulation of *Rxrs* and *Ppar* genes has been noticed in mouse models of the prodromal state of schizophrenia [60]. Interestingly, a similar effect to this observed in our present study has been seen in zebrafish embryos exposed to benzophenone-2 (BP-2), i.e., lipid droplets were accumulated in the yolk region [61].

The abovementioned results demonstrating that prenatal BP-3 administration impaired autophagy and altered RXRs and PPARγ expression levels could be at least partially related to disturbed epigenetic and post-transcriptional modifications as well as miRNAs expression. We found that embryonic offspring of dams treated with BP-3 during pregnancy exhibited decreased HDAC and sirtuin activities and a diminished level of sumoylated proteins. HDAC and sirtuin diminished activities could be responsible for RXRγ and PPARγ increased expression. Moreover, inhibition of sirtuin activity during neurodevelopment may result in inappropriate axonal differentiation, dendritic arborization and synapse formation as well as abnormal memory formation by modulating synaptic plasticity [62]. Furthermore, decreased sumoylated proteins in embryos prenatally exposed to BP-3 may be the reason for impaired autophagy, since SUMOs are modulators of chaperone-mediated autophagy (CMA) and macroautophagy [63].

Our study demonstrated that neocortical neurons derived from BP-3-exposed embryos differentially expressed 36 miRNAs which were related to neuronal development or the progression of neurological diseases. miRNAs are small noncoding RNAs that are mainly engaged in post-transcriptional mRNA regulation, and the expression levels of certain miRNAs have been postulated as biomarkers of neurological disorders [64]. In our current study, the biggest miRNA expression differences (at least 2-fold change) were noticed between the downregulation of *miR-19b*, *miR-33*, and *miR-509* and the upregulation of *miR-489*. *miR-19b* has been found to participate in neural lineage differentiation of embryonic stem cells, and the reduction in *miR-19b* expression has been recently observed in cerebrospinal fluid in AD and PD [65–69]. *miR-33* is known to inhibit cholesterol efflux and control of apoE lipidation and β-amyloid metabolism as well as stimulation of macroautophagy [70–72]. In our study, impaired autophagy and oil droplets observed during isolation of brain structures could be due to downregulation of *miR-33* that would be a prerequisite of AD. Additionally, abnormal *miR-33* and *miR-509* patterns have been connected to AD, major depression, psychosis, and anxiety disorders [73–75].

In the present study, 7-fold upregulation in *miR-489* expression could be linked to schizophrenia because high expression of this miRNA has been detected in post-mortem human brains [76]. Furthermore, in our study, BP-3-evoked alterations in miRNAs expression such as *let-7d-5p*, *let-7e-5p*, *miR-105*, *miR-107-3p*, *miR-126-5p*, *miR-152-3p*, *miR-15a-5p*, *miR-15b-5p*, *miR-195a-5p*, *miR-20b-5p*, *miR-24-3p*, *miR-26b-5p*, *miR-29a-3p*, *miR-29b-3p*, *miR-29c-3p*, *miR-302a-5p*, *miR-302b-5p*, *miR-30a-5p*, *miR-33-5p*, *miR-346-5p*, *miR-455-5p*, *miR-489-3p*, *miR-7a-5p*, and *miR-9-3p* exhibit very similar pattern to these observed in brains of schizophrenia patients [76, 77]. The strong upregulation of *miR-489* after prenatal exposure to BP-3 could suggest the inhibition of neurite overgrowth and involvement in the etiology of autism, since altered expression of *miR-489* has been found in serum of autistic children [78, 79]. We demonstrated that the expression of the remaining 32 miRNAs was also altered in response to prenatal BP-3, and these miRNAs have been related to autism, anxiety disorder, Tourette’s syndrome, AD, PD, HD, prion disease, and spinocerebellar ataxia 1.

## Conclusions

Our study revealed that prenatal exposure to BP-3 used in environmentally relevant doses impaired autophagy in terms of BECLIN-1, MAP1LC3B, autophagosomes formation, and autophagy-related factors, disrupted the levels of RXRs and PPARγ, altered epigenetic status (i.e., attenuated HDAC and sirtuin activities), inhibited post-transcriptional modifications in terms of global sumoylation, and dysregulated expression of neurogenesis- and neurotransmitter-related genes as well as miRNAs involved in pathologies of the nervous system. Our study also showed that BP-3 has good permeability through the BBB. Taking these data into account, we strongly suggest that BP-3 can significantly affect the neural development, which may be the fetal basis of the adult onset of nervous system diseases, particularly schizophrenia and AD-like neurodegenerations. Noteworthy, a recent paper by Philippat et al. demonstrated alteration in behavior of male infants in response to prenatal BP-3 exposure [80].

In our study, an involvement of prenatal exposure to BP-3 in etiology of schizophrenia is supported by impaired autophagy including lowered expression of BECLIN-1, downregulated levels of RXRα and RXRβ, elevated expression levels of neurogenesis-related factor *Mef2c*, and neurotransmitter receptors *Grin2a* and *Chrna4*, as well as by dysregulation of 24 miRNAs, particularly upregulation of *miR-489*. Less correlation is observed between the effects of prenatal exposure to BP-3 and the AD-related changes. In our study, the link between prenatal exposure to BP-3 and AD is evidenced by impaired autophagy and expression levels of RXRα and RXRβ, upregulation of apoE, and dysregulation of 26 miRNAs, mainly *miR-19b* and *miR-33*.

## Abbreviations

Actb: β-actin
AD: Alzheimer’s disease
ALS: amyotrophic lateral sclerosis
Aβ: amyloid beta
ANOVA: analysis of variance
apoE: apolipoprotein E
Atg: autophagy-related genes
BDNF: brain-derived neurotrophic factor
BP-3: benzophenone-3
BBB: blood-brain barrier
BSA: bovine serum albumin
CDC: Centers for Disease Control and Prevention
CHRNA4: cholinergic receptor nicotinic alpha 4 subunit
CMA: chaperone-mediated autophagy
CNS: central nervous system
DDE: dichlorodiphenyldichloroethylene
DIV: days in vitro
DMSO: dimethyl sulfoxide
EGF: epidermal growth factor
FBS: fetal bovine serum
FDA: Food and Drug Administration
Gapdh: glyceraldehyde-3-phosphate dehydrogenase
GDNF: glial cell-derived neurotrophic factor
GFAP: glial fibrillary acidic protein
GRIN2A: glutamate ionotropic receptor NMDA type subunit 2A
HAT: histone acetyltransferases
HDAC: histone deacetylase
Hprt: hypoxanthine-guanine phosphoribosyltransferase
HD: Huntington’s disease
MEF2C: myocyte-specific enhancer factor 2C
miRNA: microRNA
Nurr1: nuclear receptor-related 1 protein
Nurr77/NR4A1: nerve growth factor IB
*P*_app_: apparent permeability
PD: Parkinson’s disease
PPAR: peroxisome proliferator-activated receptors
qPCR: quantitative polymerase chain reaction
RAR: retinoic acid receptor
RXR: retinoid X receptors
SUMOs: small ubiquitin-like modifiers
TIC: total ion current
TOCP: tri-o-cresyl phosphate
UV: ultraviolet light
